# Can REDD+ Help the Conservation of Restricted-Range Island Species? Insights from the Endemism Hotspot of São Tomé

**DOI:** 10.1371/journal.pone.0074148

**Published:** 2013-09-16

**Authors:** Ricardo Faustino de Lima, Fábio Olmos, Martin Dallimer, Philip W. Atkinson, Jos Barlow

**Affiliations:** 1 Lancaster Environment Centre, Lancaster University, Lancaster, Lancashire, United Kingdom; 2 Associação Monte Pico, Monte Café, Mé Zóchi, República Democrática de São Tomé e Príncipe; 3 Permian Brasil, São Paulo, Brazil; 4 Center for Macroecology, Evolution and Climate, University of Copenhagen, Copenhagen, Denmark; 5 British Trust for Ornithology, The Nunnery, Thetford, United Kingdom; Institut Mediterrani d’Estudis Avançats (CSIC/UIB), Spain

## Abstract

REDD+ aims to offset greenhouse gas emissions through “Reduced Emissions from Deforestation and forest Degradation”. Some authors suggest that REDD+ can bring additional benefits for biodiversity, namely for the conservation of extinction-prone restricted-range species. Here, we assess this claim, using São Tomé Island (Democratic Republic of São Tomé and Príncipe) as a case study. We quantified the abundance of bird and tree species, and calculated the aboveground carbon stocks across a gradient of land-use intensity. We found a strong spatial congruence between carbon and the presence and abundance of endemic species, supporting the potential of REDD+ to protect these taxa. We then assessed if REDD+ could help protect the forests of São Tomé and Príncipe. To do so, we used OSIRIS simulations to predict country-level deforestation under two different REDD+ designs. These simulations showed that REDD+ could promote the loss of forests in São Tomé and Príncipe through leakage. This happened even when additional payments for biodiversity were included in the simulations, and despite São Tomé and Príncipe having the fourth highest carbon stocks per land area and the second highest biodiversity values according to the OSIRIS database. These results show weaknesses of OSIRIS as a planning tool, and demonstrate that the benefits that REDD+ might bring for biodiversity are strongly dependent on its careful implementation. We recommend that payment for ecosystem services programmes such as REDD+ develop safeguards to ensure that biodiversity co-benefits are met and perverse outcomes are avoided across all tropical countries. In particular, we advise specific safeguards regarding the conservation of extinction-prone groups, such as island restricted-range species.

## Introduction

The United Nations Conference on Environment and Development, held in Rio de Janeiro in 1992, resulted in the creation of the Convention on Biological Diversity – CBD [1] and of the United Nations Framework Convention on Climate Change – UNFCCC [2]. These legally binding treaties are regarded as landmarks towards global sustainability and were signed by most United Nation countries. However, twenty years on, these treaties remain a long way off from halting biodiversity loss and reducing emission of greenhouse gases [3].

In recent years, Reducing Emissions from Deforestation and Forest Degradation (REDD) has emerged as a key UNFCCC mechanism. This programme envisages that developed countries counterbalance their greenhouse gas emissions with incentives to reduce emissions from forest loss and degradation in developing countries. REDD had now been extended to REDD+, which includes extra mechanisms to promote sustainable forest management, conservation of existing stocks and enhancement of stocks on degraded lands [4]. As the conversion of natural areas to human use is a major contributor to climate change [5], but simultaneously the main driver of biodiversity loss [6] there are potential synergies between REDD+ mechanisms and the CBD goals [7]. In fact, to make sure that this potential is met and that negative outcomes are prevented, several biodiversity safeguards are currently being included in REDD+ investment strategies [4]. Several studies have shown that carbon stocks and biodiversity can be spatially congruent both at local and global scales [8], [9], further highlighting the potential of REDD+ to bring benefits for the conservation of biodiversity. Tropical forests are particularly relevant in this context, since they are the most biodiverse [10] and amongst the most carbon-rich [11] terrestrial ecosystems. Furthermore, they are also under threat due to the accelerated expansion of the agricultural frontier in the tropics [12].

Despite the potential of REDD+ to support biodiversity conservation, there are also concerns regarding its effectiveness [13], [14]. First, the magnitude of predicted REDD+ investments is minuscule when compared to the economic incentives currently associated with deforestation [15]. Second, REDD+ is vulnerable to leakage: by changing the availability of forest products, it might displace deforestation and forest disturbance and make some biodiversity hotspots more vulnerable to anthropogenic degradation [16], [17]. Finally, to achieve its ambitious goals and avoid perverse outcomes, REDD+ will have to be carefully translated into policy and practice [4], [18]. So far, the implementation of REDD+ remains surrounded by uncertainties that cast serious doubts about its feasibility [19], [20], [21] and few studies have tried to understand if the implementation of REDD+ mechanisms can effectively protect areas that hold important carbon stocks and high levels of biodiversity.

Restricted-range species are a priority for biodiversity conservation [24]. Their small area of occurrence makes them intrinsically vulnerable to threats, in particular if they are constrained to islands [6], [22], [23]. Buchanan et al. [25] showed that REDD+ can play an important role in the conservation of restricted-range species, but presented little evidence about how this could be put into practice. Here, we assess if REDD+ can help protect island restricted-range species, using the endemic-rich island of São Tomé as a case study. Specifically we (1) explore spatial congruence between carbon stocks and the endemics, across the island-wide gradient of land-use intensification, and (2) try to understand if REDD+ can help avoid deforestation in São Tomé. Finally, we provide some recommendations to maximise the benefits and avoid the harm that REDD+ might bring for the conservation of restricted-range island species.

## Methods

### Ethics Statement

This work was partially conducted inside the protected area of the “Parque Natural do Obô de São Tomé” [26] and involved sampling protected species, namely counting birds, and counting and measuring trees. No authorization was required for the fieldwork described here, but local authorities (the general director for the environment and the director of the protected area) were still made aware of the current study. Sampling sites were located in public land, but we asked permission to the relevant institutions when the land was privately concessioned.

### Study Area

The small oceanic islands of São Tomé and Príncipe constitute the Democratic Republic of São Tomé and Príncipe (STP, Central Africa: 0°01′08′′–0°24′27′′N, 6°27′43′′–6°45′39′′E), which holds an exceptionally high number of endemic species across a wide variety of taxa [27]. São Tomé alone holds 45 resident terrestrial bird species, of which 20 are endemic [28], and 602 spermatophyte plants, of which 96 are also endemic [29], [30]. It is estimated that old-growth forest, secondary forest and shade plantation, each cover around 30% of the island [31], [32], [33], but there are clear signs of increasing human pressure on forestry resources from land-use change and the intensification of practices within each land-use [33].

### Sampling Design

**Figure 1:**
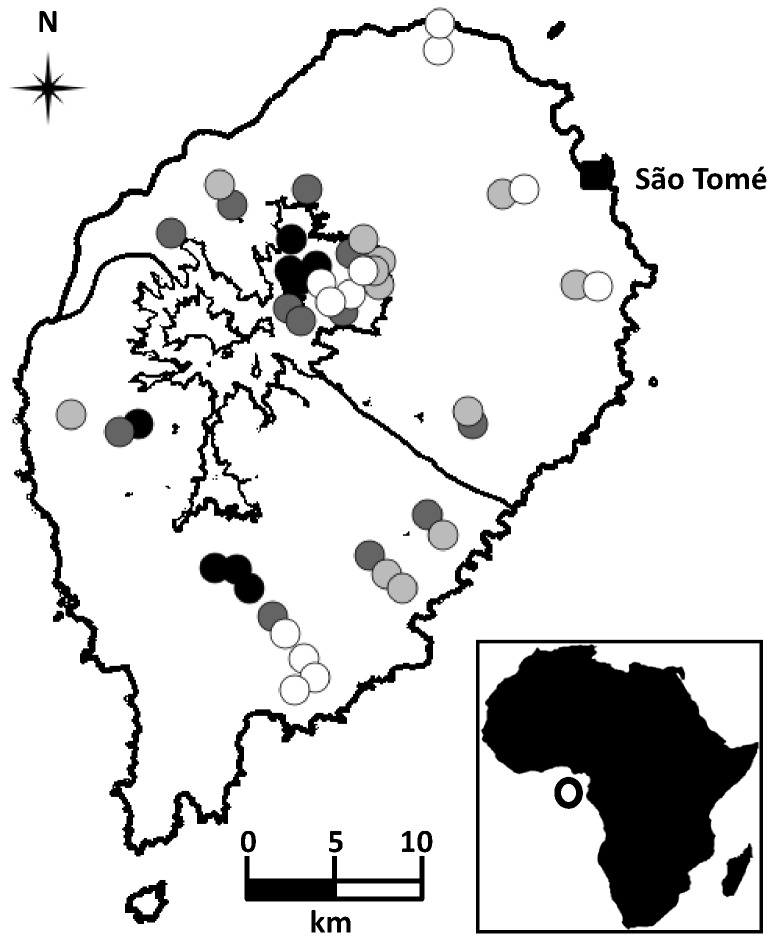
Map of São Tomé. The inset in the bottom right shows the location of the island in Africa (hollow dot). The contour lines are showing the three sampling regions: Montane (between 800 and 1400 m a.s.l.), North (up to 800 m a.s.l. and less than 2000 mm of annual rainfall) and South (up to 800 m a.s.l. and more than 3000 mm of annual rainfall). Each dot in the main map represents a transect, which is coloured according to the land-use sampled: black – old-growth forest; dark grey – secondary forest; light grey – shade plantation; and white – non-forested. The capital, São Tomé, is signed by a black square.

We divided the island into three sampling regions: montane, delimited by the 800 m and 1400 m a.s.l. altitudinal ranges, which define a distinct forest type [34]; North, comprising dry lowlands, with an annual rainfall under 2000 mm; and South, limited by the 3000 mm annual rainfall isohyet [35], [36]. Within each region, we defined four transects per main land-use category. In order to estimate the coverage of each land-use and to be able to analyse landscape context, we followed the forest inventory’s four broad land-use categories [37], [38]. These were, in order of increasing anthropogenic influence: old-growth forest, secondary forest, shade plantation and non-forested habitats. We tried to locate four replicate transects in each of the four land uses, replicated across three regions (planned n = 48), but old-growth forest in the North was too fragmented and could not be sampled effectively (Fig. 1– actual n = 44). We sampled five points within each transect, and these were separated by between 200 and 250 m (220 points in total). The coordinates of each point were taken by GPS (Garmin GMAP 76Cx).

In old-growth forest, the native flora dominates and there is no recent history of plantation or heavy logging. It is characteristic of this forest to have a high diversity of Orchidaceae and Pteridophyta, and a high proportion of endemic tree species, notably Rubiaceae and Euphorbiaceae. Invasive exotic plant species might be present (e.g.: *Cinchona ledgeriana*, *Persea americana*, *Cecropia peltata*). Secondary forests developed as a result of agricultural abandonment or tree felling for wood or charcoal. In these forests the endemic and large trees are rare, while non-endemic tree species abound, especially those that produce low-quality timber (e.g.: *Artocarpus altilis*, *Erythrina poeppigiana*, *Celtis gomphophylla*). Shade plantation refers to an agroforestry system where crops grow under the canopy of trees. The most typical of these in São Tomé are cocoa (*Theobroma cacao*) and coffee (*Coffea* sp.), which are often intercropped with banana (*Musa* sp.) and taro (*Colocasia esculenta*), and shaded by coral trees (*Erythrina* sp.) and other species, which often provide food, wood or medicine to humans. The non-forested category includes a diverse range of man-made agricultural systems in which the canopy cover is not continuous, such as oil palm monocultures, artificial savannahs and smallholder horticultures [39].

### Bird Sampling

We sampled bird assemblages during the dry seasons of 2009 and 2010 using 10-minute point counts, which were conducted between dawn and 11 am. During these counts we registered the number of individuals for each species detected within a 20 m radius. By excluding observations beyond this distance, we can maximise the independence between points and guarantee that the observations refer to birds within the land-use being sampled. Each point was sampled once by each of three experienced observers, and the bird assemblage in each point was estimated by summing these three sampling periods. This design provided a robust estimate of the bird assemblage structure, while diluting any potential influence of observer bias.

### Tree Sampling

We sampled trees within a circular plot of 0.05 ha around each point [40]. In each plot we identified all trees to the species, measured diameter at breast height (d.b.h.) with a tape measure and canopy height with a clinometer [41]. We considered ‘trees’ to include all woody plants with a d.b.h. ≥10 cm, including palms.

### Estimating Aboveground Carbon Stocks

We focused our estimation of carbon stocks on aboveground live tree carbon (AGC), since this is usually the largest component of carbon stocks in tropical forests, is the most sensitive to land-use changes and can be assessed using cost-effective standardized methods [42]. To account for errors associated with estimating AGC [43], we (1) collected data on d.b.h., height and species of each tree, to use the best available allometric equations, (2) merged data collected in plots sampled within a transect, to maximize the area sampled per data point, and (3) evaluated the representation achieved by our sampling design. We followed the conventional assumption that half of a tree’s biomass consists of carbon [44].

The aboveground biomass of each individual tree was calculated using pantropical allometric equations [44], [45] (Table 1). Even though these equations should not be used for trees outside the range size for which they were built, they remain the best available method to estimate this crucial component of total tree aboveground biomass [43]. Specific wood densities were calculated from the average wood density referenced for each species in the global wood density database [44], [46]. When species were missing in the database we calculated the average for the corresponding genus, as wood density tends to be conservative within this taxonomic level [47]. When genus was also missing from the database, we used transect-level wood density averages [44]. We estimated AGC per transect by summing the estimates obtained for each of the five 0.05 ha circular plots that composed each transect, and used this 0.25 estimate as the smallest sampling unit in all subsequent analyses. Small plots, such as the ones we used, can overestimate tree biomass [48], so we advise care when comparing with AGC estimates obtained from other studies. In terms of representation we calculate that our sampling design obtained AGC estimates for each land-use in each region with an associated error smaller than 27% at a 95% confidence interval [49].

**Table 1:** Equations used to estimate tree biomass (B).

Tree type	Forest type	Allometric equation	Reference
Palm	Any	B = e^–6.3789–0.877 ln(1/D2) +2.151 ln(h)^	[45]
Non-palm	Wet	B = 0.112 (ρD^2^h)^0.916^	[44]
Non-palm	Moist	B = 0.0509 (ρD^2^h)	[44]
Non-palm	Dry	B = 0.0776 (ρD^2^h)^0.940^	[44]

Forest types were defined for each transect based on rainfall data from [36]. In the equations, d stands for specific wood density, h for height and D for d.b.h.

### Analysing Congruence between Carbon Stocks and Biodiversity

We evaluated the congruence between AGC and species richness for birds and trees, first including all species and then just the endemics. At the transect level, we used Spearman’s rank correlation coefficient across all land-uses, and within each land-use. At the land-use level, we assessed between land-use differences in AGC using Tukey tests for linear mixed-effect models (LME), with region as a random factor. Differences in species richness were evaluated with sample-based rarefaction curves calculated in EstimateS v. 8.0 [50], [51].

To identify correlations between AGC and the abundance of each species, we calculated Spearman’s rank correlation coefficient at the transect level. We used Tukey tests for linear models to assess differences between correlation values obtained for endemic and non-endemic species. Unless stated otherwise, statistical procedures were carried out in R v. 2.10.0 [52].

### Allocation of REDD+ Funds and the Distribution of Avoided Deforestation

We used OSIRIS v.3.4 [53] to assess how the allocation of REDD+ funds would influence deforestation in São Tomé. OSIRIS was designed to support UNFCCC negotiations, and consists of a spreadsheet-based model that is linked to a database, which contains extended information on 85 countries that were considered eligible for REDD+ payments (e.g.: area, forest area, human development index, rate of deforestation, carbon stocks, human development index, number of endemic forest-dependent vertebrate species). OSIRIS assumes that prices for timber and agriculture compete with forests for the use of land in the tropics, allowing users to simulate several outcomes of REDD+ under distinct payment allocation scenarios. In our case, it allowed us to assess the effects of REDD+ on the distribution of deforestation at the country level.

We analysed how REDD+ would influence deforestation, under two scenarios. The first referred to the default values of OSIRIS, in which only payments for carbon stocks are included. The second allowed adding a payment for biodiversity value, of up to $10,000 per hectare. This biodiversity value was based on the number of nationally endemic forest-dependent amphibian, bird and mammal species [54].

Since OSIRIS is based on a country-by-country comparison, we used STP as a surrogate for São Tomé - the island represents 85% of the country’s area, and holds 20 of the country’s 27 endemic birds. We also broadened our analysis to examine how REDD+ would influence deforestation in other countries included in OSIRIS, paying special attention to those with island endemic bird species that use forest habitats. Data on endemic birds was obtained from Stattersfield et al. [24].

## Results

### Congruence between Carbon Stocks and Species Richness at the Transect Level

At the transect level, there was a significant positive correlation between AGC and species richness across land-uses for all groups of species analysed, except for total bird species richness (Table 2). Within land-uses, a positive correlation was only observed for total and endemic tree species in old-growth forest. There was a significant negative correlation between AGC and the total number of tree species in shade plantation.

**Table 2:** Correlation between AGC and observed species richness at the transect level.

	Land-use type				
Species Richness	Old	Sec	Shd	Non	All
Total bird	0.17^n.s.^	−0.31^n.s.^	−0.02^n.s.^	−0.06^n.s.^	−0.11^n.s.^
Endemic bird	0.05^n.s.^	−0.13^n.s.^	−0.01^n.s.^	0.29^n.s.^	**0.34** *
Total tree	**0.77** *	0.30^n.s.^	−**0.61** *	0.03^n.s.^	**0.50** **
Endemic tree	**0.79** *	0.03^n.s.^	0.37^n.s.^	−0.31^n.s.^	**0.51** **

Significance levels: ^n.s.^ - >0.05;

* - <0.05;

** - <0.001.

Spearman’s rank correlation coefficients for the different land-uses; old-growth forest – Old, secondary forest – Sec, shade plantation – Shd, non-forested – Non and all together – All. Corresponding significance levels are are also shown, with significant correlations (p≤0.05) evidenced in bold.

### Congruence between Carbon Stocks and Species Richness at the Land-use Level

**Figure 2:**
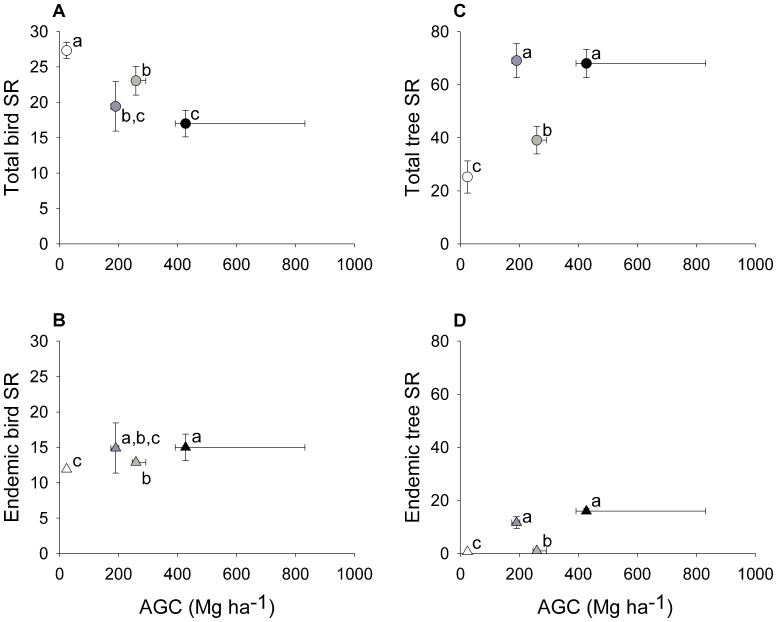
Relationships between aboveground carbon stocks (AGC) and species richness (SR) at the land-use level. The dots’ colours represent land-use; old-growth forest (black), secondary forest (dark grey), shade plantation (light grey) and non-forested (white). Horizontal bars identify the 25^th^ and 75^th^ percentile for AGC, while the vertical ones identify the 95% confidence intervals obtained from sample-based rarefaction curves. Small case letters indicate the grouping of land-uses by SR, with *a* representing the richest and *c* the poorest. The AGC was significantly higher in old-growth forest than in any other land-use, followed by shade plantation, secondary forest and non-forested land-uses, which was only not significantly smaller from secondary forest.

As expected, carbon stocks decreased with land-use intensity. The AGC was significantly higher in old-growth forest than in any other land-use, with an average of 319.8 Mg ha^−1^ (240.7 s.d.). It was followed by shade plantation with 186.0 Mg ha^−1^ (60.7 s.d.) and by secondary forest with 121.5 Mg ha^−1^ (55.7 s.d.). Non-forested land-uses had an average AGC of 15.1 Mg ha^−1^ (9.8 s.d.), which was significantly smaller than other land-uses except secondary forest (Tukey tests for LMEs with a 95% confidence interval, Figs. 2 & S1).

Species richness did not change consistently across land-uses for all groups of species analysed. Total bird species richness tended to increase in more intensive land-use types. Endemic bird richness tended to decrease, with all differences between land-use types being significant except between secondary forest and old-growth forest and between secondary forest and shade plantation. Total and endemic tree species richness also tended to decrease in more intensive land-use types, with all differences between land-use types being significant except between old-growth forest and secondary forest (Figs. 2 & S2).

At the land-use level, AGC was positively associated with species richness, except for total bird species richness (Fig. 2). Old-growth forest holds simultaneously the highest AGC and the highest number of endemic bird and tree species. Secondary forest has significantly less AGC than old-growth forest, but holds similar number of species across all groups of species considered. Shade plantation has an AGC similar to secondary forest, but holds a significantly smaller number of total and endemic tree species. It also has significantly fewer endemic bird species than old-growth forest. Non-forested land-uses have significantly fewer AGC than other land-use types except secondary forest. They also have a significantly smaller number of total and endemic tree species than other land-use types, and significantly fewer endemic bird species than old-growth forest and shade plantation. On the other hand, non-forested land-uses have a total number of bird species significantly higher than any other land-use.

### Congruence between Carbon Stocks and Species Abundance

**Figure 3:**
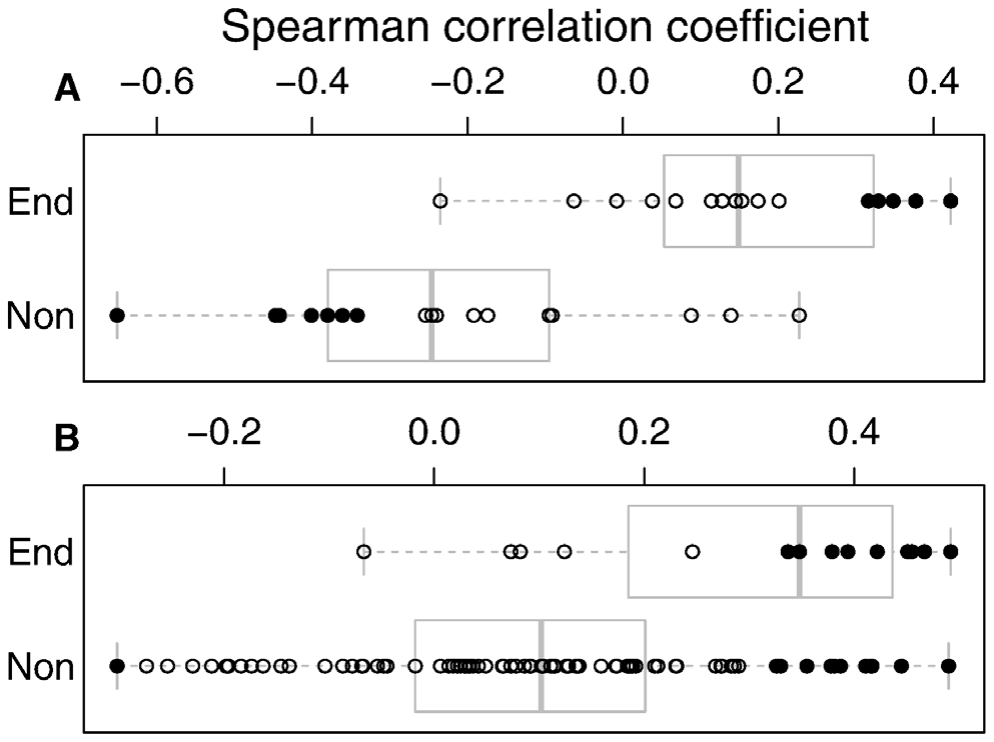
Correlation coefficient between AGC (Mg ha^−1^) and species abundance. Each circle shows the Spearman correlation coefficient value for each bird (a) or tree (b) species, at the transect level. Filled circles signal significant correlation values. Species are grouped in endemic (End) and non-endemic (Non). Boxplots show the 25^th^, 50^th^ and 75^th^ percentiles, with whiskers giving a 95% confidence interval. Note that the scales in a) are different from the ones in b).

The abundance of endemic bird and tree species tended to be positively correlated with AGC, and the correlation coefficients of endemic species were significantly higher than those between non-endemic species and AGC (Fig. 3, Tukey test for linear models with a 95% confidence interval, p<0.001). Bird species tended to have a wide range of Spearman correlation coefficient values, from −0.65 for the widespread *Estrilda astrild* (common waxbill) to 0.42 for the vulnerable endemic *Oriolus crassirostris* (São Tomé oriole). Trees showed a narrower range of coefficients, from −0.30 for the introduced *Albizia lebbeck* (lebbek) to 0.49 for the vulnerable endemic *Craterispermum montanum* (macambrará), and also tended to be more positively correlated with AGC than birds (Fig. 3).

### Allocation of REDD+ Payments, Distribution of Avoided Deforestation and Endemic-rich Countries

**Figure 4:**
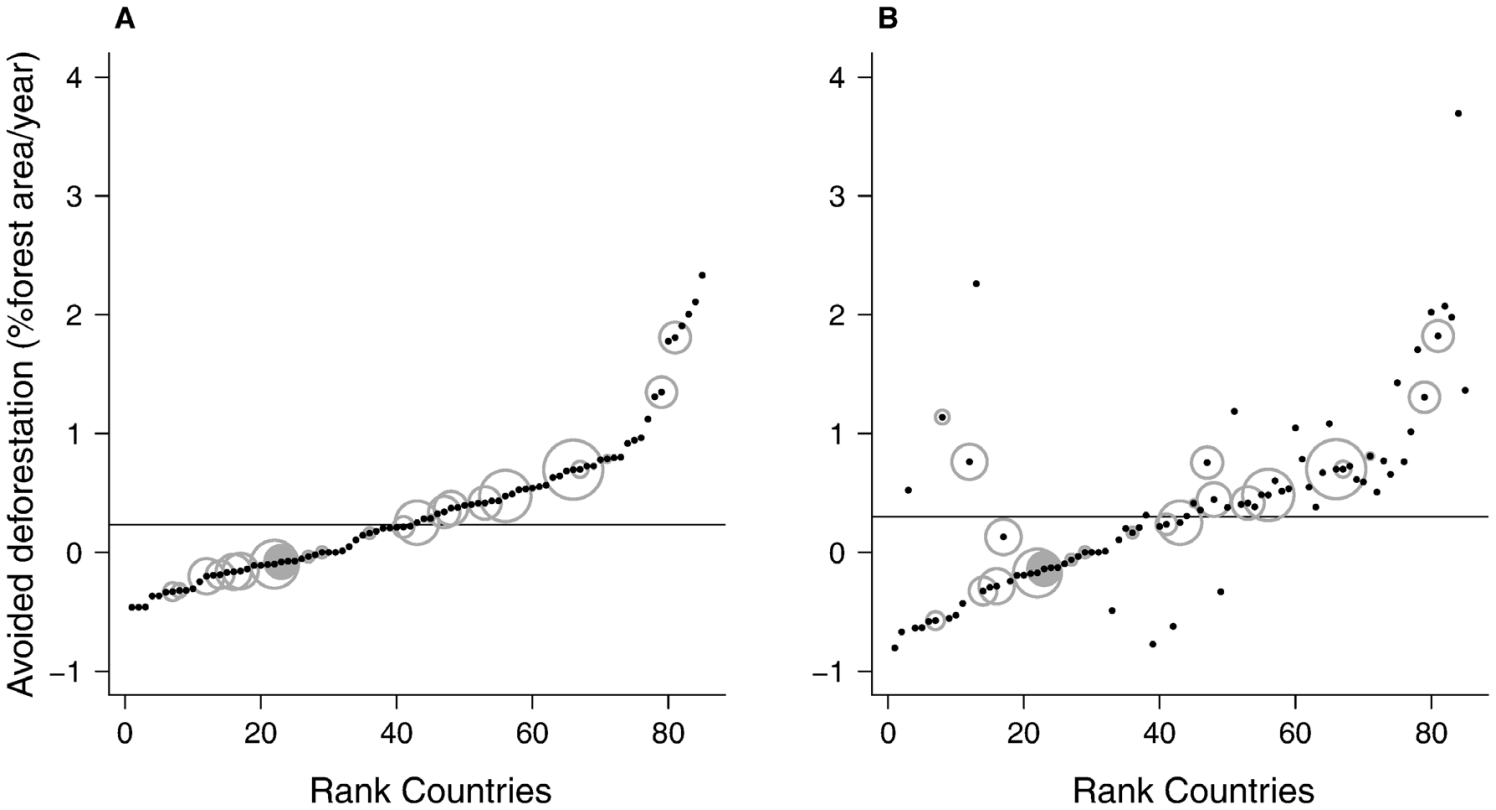
Deforestation avoided by REDD+ per country, expressed in percentage of forest area per year. Two possible scenarios are presented; a) not valuing biodiversity and b) attributing a maximum value of up to $10,000 per hectare. Countries are ranked by the percentage of avoided deforestation in the scenario a). An horizontal line signals worldwide deforestation avoided in both scenarios; 0.23 and 0.30% respectively. Grey circles show the logarithm of base 10 of the number of endemic forest bird island species for each country, with STP represented by the grey-filled circle. Negative values indicate that REDD+ is promoting deforestation, instead of avoiding.

For a scenarios with REDD+ paying for carbon stocks only, OSIRIS simulations suggest that the programme it would avoid the loss of 0.23% of forest area per year worldwide. However, this avoided deforestation would not be equally distributed across countries (Fig. 4a & Table S1), and by protecting forests in some countries, REDD+ would make other countries more vulnerable to deforestation through leakage. For example, REDD+ would promote deforestation in STP by 0.08% of the forested area per year. Ivory Coast would be the most negatively affected country, with deforestation being promoted by 0.46% of the forested area per year, while Togo would be the most benefited, with 2.33% avoided deforestation per year.

In the alternative scenario in which REDD+ includes payments for both carbon stocks and biodiversity (Fig. 4b & Table S1), OSIRIS estimated that the worldwide avoided deforestation would increase to 0.30% of the total forest area per year. Paradoxically, OSIRIS suggests that this would further increase the rate of deforestation in STP, with promoted deforestation increasing to 0.14% of the forested area per year. Under this scenario, Ivory Coast would remain as the most negatively affected country (deforestation promoted by 0.80%), while Nigeria would benefit the most, with avoided deforestation increasing from 2.11 to 3.70% of the forested area per year.

The difference in avoided deforestation for the two abovementioned scenarios also varied markedly between countries (Table S1). Overall, the inclusion of payments for biodiversity would lower avoided deforestation for most countries (44 out of 85), while a few would benefit greatly. Chad registered the highest decrease in avoided deforestation, when comparing the two scenarios (from 0.20% in the default to −0.77% when biodiversity payments were included), while Pakistan registered the highest increase (from −0.19 to 2.26%). The same trend was observed amongst countries rich in forest endemic island bird species. Although STP would be negatively affected by the inclusion of biodiversity payments in REDD+(from −0.08 to −0.14), other island countries such as Haiti, which holds 34 forest endemic island birds, would be benefited (from −0.20 to 0.76%). Avoided deforestation changed little for the countries with more forest endemic island birds (Table 2), such as Indonesia (373 species), Papua New Guinea (170) and the Philippines (120).

## Discussion

### Spatial Congruence between Carbon Stocks and Endemic Biodiversity

The abundance of most species showed a positive correlation with forest carbon stocks. Most exceptions were non-endemics, notably birds, with the endemic species showing an overall more positive association with carbon-rich areas than the non-endemic species. These findings are not surprising, considering that São Tomé was entirely forested when it was found by the Portuguese in 1471 and that many of the species now occupying degraded habitats are thought to have been introduced [32]. The potential benefits of REDD+ in STP are obvious, not only due to the importance of its forests to storage carbon and to protect biodiversity, but also to ensure a sustainable management of marketable forest resources, like timber [57]. In particular, secondary forests are good candidates for the development of biodiversity-friendly carbon enhancement projects, since this land-use type has severely depleted carbon stocks but can still hold a significant number of endemic species (Fig. 2).

Forests worldwide hold a significant proportion of terrestrial biodiversity [10], including many endemic species. According to Stattersfield et al. [24], 2306 of all restricted-range bird species (88% of total) occur in forests. Therefore, it is likely that the link between forest carbon stocks and endemic species holds in many other endemic bird areas around the globe, and that, by funding the protection of forest carbon stocks, REDD+ can make a significant contribution to the conservation of these extinction-prone taxa.

### Allocation of REDD+ Payments and Avoided Deforestation in Endemic-rich Countries: The Devil in the Detail

The potential of REDD+ to help the conservation of endemic species [25] will only become a reality if the territories holding those species have access to REDD+ payments. According to OSIRIS [53], the implementation of payments for carbon stocks through REDD+ would avoid the loss of 0.23% of the total forest area per year worldwide, but leakage would promote deforestation in some countries. The response of countries rich in forest endemic island bird species to REDD+ was also varied; avoided deforestation was predicted to increase in some and decrease in others (Fig. 4A & Table S1). Worldwide deforestation was predicted to decrease even further when payments for biodiversity were included in REDD+, but again the response was highly variable between countries. In some countries REDD+ would start protecting forests, while in others, such as the extremely endemic-rich STP, it would promote yet further deforestation (Fig. 4 & Table S1).

According to the OSIRIS database, STP has, per area, the fourth highest carbon stock and the second highest biodiversity value of countries able to receive payments under REDD+ [53]. It is therefore surprising that OSIRIS predicts that REDD+ will promote deforestation in STP, and that this deforestation will be promoted even further if biodiversity value is taken into account. This outcome is difficult to interpret due to the complexity of the model underlying OSIRIS. It is nevertheless particularly worrying that including biodiversity payments in REDD+ will decrease the avoided deforestation in the endemic-rich STP even further, when OSIRIS biodiversity value is based on the number of nationally endemic forest-dependent vertebrate species. Our results raise concerns about how OSIRIS’ underlying model is weighting each component to define investment priorities and to predict leakage-induced deforestation. Some of these surprising results may be explained by inaccuracies and uncertainties within OSIRIS. According to its database, STP has 270 km^2^ of forest, no deforestation and only two nationally endemic forest-dependent amphibian, bird and mammal species. Other sources indicate 284 km^2^ of primary forest and 301 of secondary forest (plus 323 of agroforestry) [32], clear signs of deforestation and forest degradation [33] and at least six (and up to 16) nationally endemic forest-dependent amphibian, bird and mammal species [55] (Table S2). Adopting the latter values should have major implications for the estimates of avoided deforestation in STP, unless similar changes are also to be made for other countries included in the OSIRIS database.

It is also worth noticing that many island endemic-rich countries (e.g.: Fiji, with 36 island endemic bird species, and nearly all other countries from Oceania) and territories (e.g. New Caledonia or Hawaii, both with 31 island endemic bird species) are not listed in OSIRIS. This tool only included countries thought to be potentially eligible for REDD+ [53], [54], so it is justified that territories are not included, as they belong to developed countries (e.g. New Caledonia to France or Hawaii to the U.S.A.). However, the choice of countries to be included in the OSIRIS database is less clear; Why were Cuba and Brazil included, while the less developed Fiji and Comoros were left out? Regardless of the reasons behind these choices, it is worrying that these areas were not considered eligible for REDD+ mechanisms, as this would make them especially vulnerable to deforestation through leakage.

### Making REDD+ Work for the Conservation of the Endemics

The success of REDD+ for protecting restricted-range island endemic species will ultimately depend on its implementation, a process that remains surrounded by uncertainties [21]. Despite specifically valuing endemic-rich forests, REDD+ payments based on decisions supported by OSIRIS would still make some of these areas vulnerable to deforestation. Therefore, we advise extreme care in the use of global systematic approaches to define priorities for REDD+ investments. Failing to acknowledge the constraints of these approaches might have serious consequences and could even promote the loss of areas that are extremely rich in both carbon and biodiversity.

Forests holding restricted-range island species are often small and species-poor, making them easy to be overlooked in global biodiversity analysis [56]. Furthermore, their small size means they also hold a very small proportion of the world’s terrestrial carbon stocks, and are therefore easily overlooked in large-scale REDD+ prioritisation exercises, even if they are carbon-rich on a carbon stock per area basis. As the emission caps’ carbon market is heavily reliant on global systematic approaches, it will be difficult to adapt it to help protect endemic-rich island forests. We believe that this could be achieved more easily through voluntary schemes, which are more flexible and would allow the additional co-benefits for biodiversity conservation to be promoted directly to any potential investors. Our results indicate the need to develop specific conservation safeguards within REDD+ that ensure the effective protection of island restricted-range species. These safeguards should extend to similar programmes paying for ecosystem services to guarantee that potential synergies with biodiversity conservation will be met.

## Supporting Information

Figure S1 **Aboveground carbon stocks across land-uses.**
(DOCX)

Figure S2 **Sample-based rarefaction curves for birds (a,b) and trees (c,d) across land-uses.**
(DOCX)

Table S1 **Avoided deforestation according to two scenarios simulated in OSIRIS.**
(DOC)

Table S2 **São Tomé and Príncipe’s nationally endemic forest-dependent species.**
(DOC)
