# Correction: Can REDD+ Help the Conservation of Restricted-Range Island Species? Insights from the Endemism Hotspot of São Tomé

**DOI:** 10.1371/annotation/f3b7b8dc-a2d6-43e8-945a-566a7082cb12

**Published:** 2013-09-24

**Authors:** Ricardo Faustino de Lima, Fábio Olmos, Martin Dallimer, Philip W. Atkinson, Jos Barlow

The correct affiliation for the third author, Martin Dallimer, should be "Department of Food and Resource Economics, Center for Macroecology, Evolution and Climate, University of Copenhagen, Denmark" 

