# Supplementary material for: Can REDD+ Help the Conservation of Restricted-Range Island Species? Insights from the Endemism Hotspot of São Tomé
Source: PLoS One. 2013 Sep 16;8(9):e74148. doi: 10.1371/journal.pone.0074148 (PMC3774614; doi:10.1371/journal.pone.0074148)
Supplement: Figure S1 — Aboveground carbon stocks across land-uses. (DOCX) [file pone.0074148.s001.docx]

**Figure S1 – Aboveground carbon stocks across land-uses.** Each transect is shown, with shape signalling region; circle for montane, square for north and diamond for south. Boxplots show the 10^th^, 25^th^, 50^th^, 75^th^ and 90^th^ percentiles. Small case letters show land-uses grouped according to AGC, as obtained with Tukey test for LMEs with a 95% confidence interval. Land-use types are old-growth forest (old), secondary forest (sec), shade plantation (shd) and non-forested (non).
