# Supplementary material for: Can REDD+ Help the Conservation of Restricted-Range Island Species? Insights from the Endemism Hotspot of São Tomé
Source: PLoS One. 2013 Sep 16;8(9):e74148. doi: 10.1371/journal.pone.0074148 (PMC3774614; doi:10.1371/journal.pone.0074148)

**Figure S2 – Sample-based rarefaction curves for birds (a,b) and trees (c,d) across land-uses.** Small-dotted lines show the 95% confidence intervals. The graphs on top (a,c) show the total number of species, while the ones on the bottom (b,d) show just the endemic.


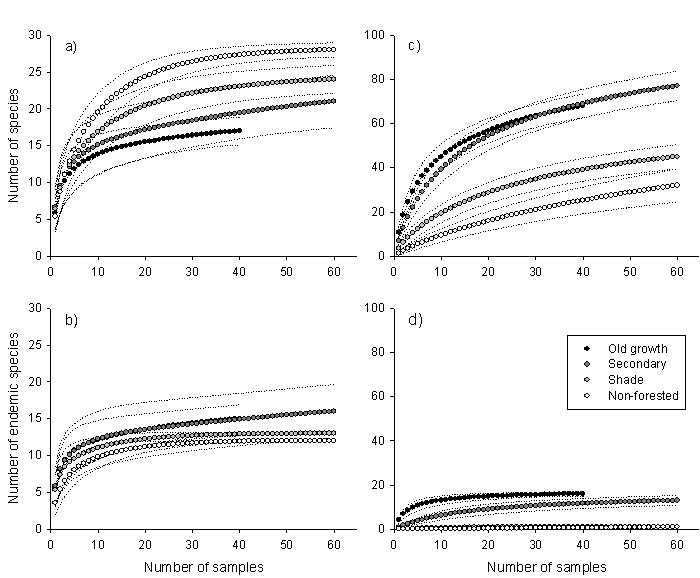

Supplement: Figure S2 — Sample-based rarefaction curves for birds (a,b) and trees (c,d) across land-uses. (DOCX) [file pone.0074148.s002.docx]
