# Supplementary material for: Can REDD+ Help the Conservation of Restricted-Range Island Species? Insights from the Endemism Hotspot of São Tomé
Source: PLoS One. 2013 Sep 16;8(9):e74148. doi: 10.1371/journal.pone.0074148 (PMC3774614; doi:10.1371/journal.pone.0074148)
Supplement: Table S1 — Avoided deforestation according to two scenarios simulated in OSIRIS. (DOC) [file pone.0074148.s003.doc]

**Table S1 – Avoided deforestation according to two scenarios simulated in OSIRIS.** In scenario 1 REDD+ includes only payments for carbon stocks, while in scenario it includes an additional payment for biodiversity value. Value of avoided deforestation are expressed as percentage of forest area per year.

| Country | Number of forest island endemic bird species | Avoided deforestation | | |
| --- | --- | --- | --- | --- |
|  |  | Scenario 1 | Scenario 2 | Difference |
| Ivory Coast | 0 | -0.46 | -0.80 | -0.34 |
| Central African Republic | 0 | -0.46 | -0.67 | -0.21 |
| Bolivia | 0 | -0.46 | 0.52 | 0.98 |
| Burkina Faso | 0 | -0.37 | -0.64 | -0.27 |
| Uruguay | 0 | -0.37 | -0.63 | -0.27 |
| Suriname | 0 | -0.33 | -0.58 | -0.25 |
| Cuba | 6 | -0.33 | -0.57 | -0.24 |
| Tanzania | 4 | -0.32 | 1.14 | 1.46 |
| Guyana | 0 | -0.32 | -0.56 | -0.24 |
| Gabon | 0 | -0.31 | -0.53 | -0.22 |
| Bangladesh | 0 | -0.25 | -0.43 | -0.18 |
| Haiti | 34 | -0.20 | 0.76 | 0.96 |
| Pakistan | 0 | -0.19 | 2.26 | 2.45 |
| India | 16 | -0.19 | -0.33 | -0.14 |
| Swaziland | 0 | -0.17 | -0.30 | -0.13 |
| Dominican Republic | 34 | -0.16 | -0.29 | -0.12 |
| Jamaica | 35 | -0.16 | 0.13 | 0.29 |
| South Africa | 0 | -0.14 | -0.24 | -0.10 |
| Iran | 0 | -0.11 | -0.19 | -0.08 |
| Vietnam | 0 | -0.11 | -0.19 | -0.08 |
| Afghanistan | 0 | -0.10 | -0.18 | -0.08 |
| Philippines | 120 | -0.10 | -0.17 | -0.07 |
| São Tomé and Príncipe | 27 | -0.08 | -0.14 | -0.06 |
| China | 4 | -0.07 | -0.13 | -0.05 |
| Lesotho | 0 | -0.07 | -0.13 | -0.05 |
| Rwanda | 0 | -0.06 | -0.10 | -0.04 |
| Chile | 3 | -0.04 | -0.06 | -0.03 |
| Bhutan | 0 | -0.02 | -0.04 | -0.01 |
| Costa Rica | 3 | 0.00 | 0.00 | 0.00 |
| Nepal | 0 | 0.00 | 0.00 | 0.00 |
| Thailand | 0 | 0.00 | 0.00 | 0.00 |
| Sudan | 0 | 0.01 | 0.01 | 0.00 |
| Congo | 0 | 0.05 | -0.49 | -0.54 |
| Republic of Korea | 0 | 0.10 | 0.11 | 0.00 |
| Democratic Republic of the Congo | 0 | 0.14 | 0.20 | 0.06 |
| Colombia | 3 | 0.16 | 0.17 | 0.00 |
| Angola | 0 | 0.18 | 0.21 | 0.03 |
| Kenya | 0 | 0.20 | 0.31 | 0.11 |
| Chad | 0 | 0.20 | -0.77 | -0.98 |
| Peru | 0 | 0.21 | 0.22 | 0.01 |
| Mexico | 8 | 0.21 | 0.24 | 0.02 |
| Mozambique | 0 | 0.22 | -0.62 | -0.84 |
| Solomon Islands | 78 | 0.25 | 0.25 | 0.00 |
| Zambia | 0 | 0.28 | 0.31 | 0.02 |
| Brazil | 2 | 0.29 | 0.41 | 0.13 |
| Panama | 1 | 0.32 | 0.36 | 0.03 |
| Sri Lanka | 23 | 0.34 | 0.76 | 0.42 |
| Madagascar | 29 | 0.37 | 0.44 | 0.07 |
| Namibia | 0 | 0.40 | -0.33 | -0.71 |
| Belize | 0 | 0.40 | 0.38 | -0.02 |
| Cambodia | 0 | 0.41 | 1.19 | 0.78 |
| Guinea-Bissau | 0 | 0.42 | 0.40 | -0.01 |
| Malaysia | 26 | 0.43 | 0.42 | 0.00 |
| Senegal | 0 | 0.43 | 0.38 | -0.05 |
| Laos | 0 | 0.47 | 0.48 | 0.05 |
| Papua New Guinea | 170 | 0.49 | 0.48 | 0.01 |
| Venezuela | 0 | 0.53 | 0.60 | 0.11 |
| Mali | 0 | 0.53 | 0.51 | -0.01 |
| Guinea | 0 | 0.54 | 0.54 | 0.00 |
| Cameroon | 0 | 0.54 | 1.04 | 0.51 |
| Argentina | 0 | 0.55 | 0.78 | 0.23 |
| Sierra Leone | 0 | 0.56 | 0.55 | -0.02 |
| Benin | 0 | 0.63 | 0.38 | -0.25 |
| Liberia | 0 | 0.64 | 0.67 | 0.03 |
| Ethiopia | 0 | 0.68 | 1.08 | 0.40 |
| Indonesia | 373 | 0.70 | 0.70 | 0.00 |
| Equatorial Guinea | 5 | 0.70 | 0.70 | 0.00 |
| Mongolia | 0 | 0.73 | 0.73 | 0.00 |
| Malawi | 0 | 0.73 | 0.61 | -0.11 |
| Botswana | 0 | 0.78 | 0.59 | -0.19 |
| Myanmar | 2 | 0.79 | 0.81 | 0.02 |
| Burundi | 0 | 0.80 | 0.51 | -0.29 |
| Paraguay | 0 | 0.80 | 0.77 | -0.03 |
| Ghana | 0 | 0.92 | 0.66 | -0.26 |
| Guatemala | 0 | 0.94 | 1.43 | 0.48 |
| Uganda | 0 | 0.96 | 0.76 | -0.20 |
| El Salvador | 0 | 1.12 | 1.02 | -0.11 |
| Zimbabwe | 0 | 1.31 | 1.70 | 0.39 |
| Timor-Leste | 21 | 1.35 | 1.31 | -0.04 |
| Nicaragua | 0 | 1.78 | 2.02 | 0.24 |
| Ecuador | 22 | 1.81 | 1.82 | 0.01 |
| Honduras | 0 | 1.91 | 2.07 | 0.17 |
| Democratic People's Republic of Korea | 0 | 2.00 | 1.98 | -0.02 |
| Nigeria | 0 | 2.11 | 3.69 | 1.59 |
| Togo | 0 | 2.33 | 1.36 | -0.97 |
