# Supplementary material for: Can REDD+ Help the Conservation of Restricted-Range Island Species? Insights from the Endemism Hotspot of São Tomé
Source: PLoS One. 2013 Sep 16;8(9):e74148. doi: 10.1371/journal.pone.0074148 (PMC3774614; doi:10.1371/journal.pone.0074148)
Supplement: Table S2 — São Tomé and Príncipe’s nationally endemic forest-dependent species. (DOC) [file pone.0074148.s004.doc]

**Table S2 – São Tomé and Príncipe’s nationally endemic forest-dependent species.** All endemic amphibian, bird and mammal species are listed and classified according to habitat information in IUCN [55]; forest-dependent if observations are restricted to forest (excluding agroforestry, such as shade plantations), probably forest-dependent if most observations are in forest or near forest and not forest-dependent, otherwise.

| Species | Class | IUCN habitat description | Forest-dependent |
| --- | --- | --- | --- |
| *Amaurocichla bocagei* | Aves | This species appears quite common in humid forest, possibly preferring boulder strewn micro-habitats where it forages for invertebrates on rocks, moss-covered stones and fallen branches. It inhabits the undergrowth of lowland riparian forest, up to over 1,000 m. Recently, it was observed flying up to the mid-storey in ridgetop forest. | Yes |
| *Bostrychia bocagei* | Aves | The species is confined to primary forest below 450 m, although suitable habitat could extend up to 800 m. However, it has been found most frequently in recent years at the border between primary and secondary forest, sometimes near palm plantations. | Yes |
| *Columba thomensis* | Aves | It is most common in primary forest above 1,000 m but also occurs in mature secondary forest, particularly coffee plantations abandoned for more than 20 years, and in lowland primary forest in the south-west and more occasionally in cultivated areas at forest edge. | Probably |
| *Crocidura thomensis* | Mammalia | C. thomensis is associated with montane tropical moist forest. | Probably |
| *Hipposideros thomensis* | Mammalia | This species is found in lowland tropical moist forest species (both primary and secondary), and can be observed in modified habitats such as plantations. It roosts in caves, lava tubes, water extraction tubes and similar structures. | Probably |
| *Horizorhinus dohrni* | Aves | Terrestrial | No |
| *Hyperolius molleri* | Amphibia | It lives in primary forest, farm bush (heavily degraded former forest), coconut groves, coffee plantations and disturbed areas. It lays its eggs on leaf surfaces over still or very slow-moving water, and the tadpoles develop in the water. | No |
| *Hyperolius thomensis* | Amphibia | It is restricted to primary rainforest, and is known to breed by larval development in tree holes. It has previously been reported from towns, but there is now significant doubt about these records. | Probably |
| *Lanius newtoni* | Aves | All records are from primary lowland and mid-altitude forest up to c.1,400 m, in sites with little or no undergrowth, but with bare ground and rocks. | Yes |
| *Leptopelis palmatus* | Amphibia | It generally inhabits wet forest along the edges of creeks and streams. It can also be found in forest remnants and possibly in towns. | Probably |
| *Miniopterus newtoni* | Mammalia | The species appears to be quite common both in forest and plantations. | No |
| *Myonycteris brachycephala* | Mammalia | This species has been recorded from montane and steep rocky, and steep sided areas. Animals have been reported from montane tropical moist forest (at 1,300 m asl), tropical lowland forest (a specimen from natural forest at 800 m asl), from an area with remnants of original vegetation (just north of Pico de São Tomé), and from cocoa plantations. The species appears to prefer the forested mountain zones but may be found to live in plantation areas and avoids the coastal zone and the northern dry part of the island. | Probably |
| *Nectarinia hartlaubi* | Aves | Terrestrial | No |
| *Nectarinia newtoni* | Aves | Terrestrial | No |
| *Nectarinia thomensis* | Aves | It occurs in both lowland and montane primary forest up to at least 2,000 m, with occasional records from forest-edge cultivation. | Probably |
| *Neospiza concolor* | Aves | It occurs in lowland, closed-canopy primary forest. | Yes |
| *Oriolus crassirostris* | Aves | It is most abundant in primary forest (up to 1,600 m), but also occurs in undisturbed secondary forest. It occurs occasionally in dry forest in the north but is generally absent from cocoa plantations. | Probably |
| *Otus hartlaubi* | Aves | It occurs in primary and undisturbed secondary forest up to 1,500 m, but not in plantations with shade trees. | Yes |
| *Phrynobatrachus dispar* | Amphibia | Present in primary forest, farm bush (heavily degraded former forest), and abandoned plantations where wet conditions prevail. | No |
| *Phrynobatrachus leveleve* | Amphibia | Present in primary forest, farm bush (heavily degraded former forest), and abandoned plantations where wet conditions prevail. | No |
| *Ploceus grandis* | Aves | Terrestrial | No |
| *Ploceus princeps* | Aves | Terrestrial | No |
| *Ploceus sanctithomae* | Aves | Terrestrial | No |
| *Prinia molleri* | Aves | Terrestrial | No |
| *Schistometopum thomensis* | Amphibia | It lives in soil in all habitats on São Tomé (except dry areas in the north), including coconut plantations, forests, trash piles, sugar cane, degraded areas, rocky areas, and totally denuded land coastal. | No |
| *Serinus rufobrunneus* | Aves | Terrestrial | No |
| *Speirops leucophoeus* | Aves | It occurs in all forested habitats, including plantations, up to 800 m. | No |
| *Speirops lugubris* | Aves | Terrestrial | No |
| *Tadarida tomensis* | Mammalia | This species has been recorded from Praia das Conchas which is quite open and the driest point of the island (open savanna forest with baobabs), and from Agua Izé which is much more humid, and although it is now cocoa plantations it was previously forest. | No |
| *Terpsiphone atrochalybeia* | Aves | Terrestrial | No |
| *Treron sanctithomae* | Aves | It occurs in primary and secondary tropical and subtropical forest, tolerating forest fragmentation and also occurring in plantations, and is found from sea-level to 1,600 m but is more common above 300 m. | Probably |
| *Turdus olivaceofuscus* | Aves | The species inhabits primary and secondary forest up to 2,024 m. It prefers lowland habitats, and also occupies cocoa plantations with *Erythrina* shade trees, orchards, gardens and coffee plantations, as well as dry woodland in savanna and cloudforest. | No |
| *Turdus xanthorhynchus* | Aves | The species has been recorded in primary forest from the lowlands to c.800 m at least, although most birds occur above 400 m. | Yes |
| *Zoonavena thomensis* | Aves | Terrestrial | No |
| *Zosterops ficedulinus* | Aves | On São Tomé, it prefers mid- to high-altitude forest up to 1,600 m, but also occurs in the lowlands. It utilises primary forest, old secondary growth and shade-forest in former cocoa plantations. The subspecies on Príncipe is confined to primary forest and forest edge. | Probably |
